# First Detection of Microcystin-LR in the Amazon River at the Drinking Water Treatment Plant of the Municipality of Macapá, Brazil

**DOI:** 10.3390/toxins11110669

**Published:** 2019-11-15

**Authors:** Elane D.C. Oliveira, Raquel Castelo-Branco, Luis Silva, Natalina Silva, Joana Azevedo, Vitor Vasconcelos, Silvia Faustino, Alan Cunha

**Affiliations:** 1 Institute of Scientific and Technological Research of the State of Amapá, Macapá, 68.903-197 Amapá, Brazilluis.abdon13@gmail.com (L.S.); natalina.borges@outlook.com.br (N.S.); 2 Bionorte Post-Graduate Program, UNIFAP, Federal University of Amapá, Macapá, 68903-419 Amapá, Brazil; fitomathes@yahoo.com (S.F.); alancunha12@gmail.com (A.C.); 3 Interdisciplinary Centre of Marine and Environmental Research, University of Porto, Terminal de Cruzeiros do Porto de Leixões, 4050-208 Matosinhos, Portugal; raquel.castelobranco.12@gmail.com (R.C.-B.); joana_passo@hotmail.com (J.A.); 4 Department of Biology, Faculty of Sciences of University of Porto, Rua do Campo Alegre, 4069-007 Porto, Portugal

**Keywords:** DWTP, ELISA, LC/MS, *Limnothrix planctonica*, *mcyE*, microcystin, toxicity, water supply

## Abstract

Human poisoning by microcystin has been recorded in many countries, including Brazil, where fatal cases have already occurred. The Amazon River is the main source of drinking water in municipalities such as Macapá, where there is no monitoring of cyanobacteria and cyanotoxins. This study investigated the presence of cyanobacteria and cyanotoxins in samples from a drinking water treatment plant (DWTP) that catches water from the Amazon River. The toxin analyses employed ELISA, LC/MS, and molecular screening for genes involved in the production of cyanotoxins. The sampling was carried out monthly from April 2015 to April 2016 at the intake (raw water) and exit (treated water) of the DWTP. This study reports the first detection of microcystin-LR (MC-LR) in the Amazon River, the world’s largest river, and in its treated water destined for drinking water purposes in Macapá, Brazil. The cyanobacterial density and MC-LR concentration were both low during the year. However, *Limnothrix planctonica* showed a density peak (± 900 cells mL^−1^) in the quarter of June–August 2015, when MC-LR was registered (2.1 µg L^−1^). Statistical analyses indicate that *L. planctonica* may produce the microcystin.

## 1. Introduction

Cyanobacteria are photosynthetic bacteria widely adapted and distributed in almost all environments. They act as primary producers in the aquatic environments, providing oxygen and energy to the ecosystem [1]. These microorganisms produce secondary metabolites of great biotechnological and industrial interest with anticancer, antiviral, anti-inflammatory, photoprotector, and insecticidal activities, among others [2,3]. On the other hand, cyanobacteria may also produce secondary metabolites that may cause harm to human health, mammals, and other organisms known as cyanotoxins [4,5].

The cyanotoxins can be classified according to their toxicity target: neurotoxins, hepatotoxins [4], cytotoxins [6], and dermatotoxins [7]. The most common are the microcystins (MC), a type of hepatotoxin that has a cyclic peptide structure, being stable to chemical hydrolysis and oxidation [8,9]. Microcystins show several variants of its basic structure, a heptapeptide ring, with more than 240 MC variants [10]. The best known and most toxic structural variant is microcystin-LR (MC-LR) [11], a variant with leucine and arginine residues at amino acid positions 2 and 4 that show high variability [12].

Microcystins are resistant to high temperatures, their toxicity remains even after boiling, and they may lead to a higher incidence of liver cancer [13,14]. Although toxic to the liver, microcystins may induce necrosis and/or apoptosis in cells from other organs than liver. In vitro studies have demonstrated the cytotoxic effects of microcystin-LR on human cells such as erythrocytes, lymphocytes, endothelial cells, epithelial cells, and fibroblasts [15,16,17].

Records of microcystin poisoning have been documented in many countries, including Brazil. In 1996, approximately 52 chronic renal patients died after undergoing hemodialysis sessions at a clinic in the city of Caruaru (PE), as the water used to treat the patients was supplied by a reservoir which was undergoing through a cyanobacteria bloom and had its water contaminated with microcystins-LR, -YR, and -AR [18]. One of the main pathways of cyanotoxin poisoning is through oral water consumption, so there is a growing concern about water supply sources, which need proper monitoring to avoid the toxic effects of these toxins.

The increase in cyanobacterial populations is indicative of cyanotoxin production risks in drinking water catchment areas. For this reason, the World Health Organization (WHO) recommends monthly monitoring of raw water with the identification of cyanobacterial genera and cell counts to predict and control cyanobacterial growth in drinking water sources [5,19]. The WHO publishes the “Guidelines for Drinking-water Quality” which brings together scientific knowledge on the quality of drinking water and disseminates it for each country to create their own standards and regulations [19].

The Brazilian regulation [20] follows the WHO recommendations and requires water supply companies to monitor cyanobacteria and cyanotoxins. Also, the Brazilian Law [20] establishes the same guideline value (GV) of WHO for microcystins of 1.0 µg L^−1^ of the intracellular + extracellular fractions [5,19]. However, such monitoring is not performed in most of the Brazilian Amazon Region, as in the Amapá State [21]. This may be due to the low surveillance of local authorities or/and the belief that the Amazon River has high self-purification power; in addition, there are few experts in the field of cyanobacteria research in the region. For all these reasons, there are no studies available on cyanobacterial density and cyanotoxin production in Amapá. Even cyanobacterial taxonomic studies are scarce [22].

This study was performed in the drinking water catchment zone of Macapá, the capital city of Amapá State, in Brazil, which is one of the populated cities located on the banks of the Amazon River, nearby its mouth. The Amazon River is the primary source of drinking water in Macapá. The raw water intake is 500 m from the river margin in the city downtown, a densely populated area where it is possible to observe punctual and diffuse pollution sources. Previous studies showed that pollutant plumes of the Amazon River in this stretch reach a distance of approximately 800 m from the river margin during the tidal cycle, but the drinking water treatment catchment point is only 500 m away from the margin [23,24]. These sources of pollution may provide nutrients that can stimulate cyanobacterial density increase and consequently increase the toxin production risk.

Cyanobacteria and cyanotoxins (especially microcystin) are common problems for drinking water treatment. Microcystins are endotoxins [25] and usually remain within cyanobacterial cells until released by cell lysis, body senescence, or other factors. Notably, in drinking water treatments, the use of cyanobacterial-killing substances may promote the release of these difficult-to-remove endocellular toxins [7]. Therefore, raw water should be monitored, and the treatment should be changed under bloom conditions, avoiding the use of chlorine [26] and algaecides such as copper sulfate [5]. Cells must be removed intact to avoid the potential release of microcystins during the water treatment processes [27]. Physical stimuli that occur during treatment may also promote cell permeabilization or breakage [28].

There is a need to understand better the cyanobacteria presence in the Amazon River freshwater to ensure that the quality of this water will remain suitable for the present and future generations. The Amazon River is the largest river on Earth, with average annual discharge at the river mouth of approximately 219 m^3^ s^−1^, representing an average of 16% to 20% of the global freshwater discharge to the ocean [29].

The goal of this study is (1) to investigate the occurrence of cyanotoxin and cyanobacterial dynamics in water from the Amazon River intended for drinking water treatment and (2) to verify the environmental factors that may induce increases in cyanotoxin production and cyanobacterial density in this area.

## 2. Results

### 2.1. Cyanotoxins

To our knowledge, this study is the first to detect the presence of MC-LR in the Amazon River waters before and after treatment for drinking water purposes. Methods were used to identify and quantify the cyanotoxins microcystin, saxitoxin, anatoxin, and cylindrospermopsin, but only MC-LR was detected in the study samples; details are presented in the following sections.

#### 2.1.1. Quantification of Cyanotoxins by ELISA

ELISA detected MC-LR in June, July, and August raw water (1.5 µg L^−1^, 2.1 µg L^−1^, and 0.6 µg L^−1^, respectively), and in June and July treated water (both 0.1 µg L^−1^). In the other months, there were very low concentrations of microcystin (0.1 µg L^−1^) in the raw water samples, with no microcystin in the treated water. The low concentrations were considered false positives, but all samples were further analyzed by molecular screening for genes involved in the production of the cyanotoxins microcystin, saxitoxin, anatoxin, and cylindrospermopsin [30].

The previously presented microcystin values were the sum of dissolved and cellular toxins. Through the separation of intracellular and extracellular fractions (Figure S1, Supplementary Materials), most of the MC-LR in the Amazon River was verified as intracellular. The highest MC-LR value occurred in July 2015, with 1.9 µg L^−1^ of intracellular MC-LR and 0.2 µg L^−1^ of dissolved-fraction MC-LR.

#### 2.1.2. Molecular Screening for Cyanotoxins

Of all the genes investigated in this study that are involved in the production of cyanotoxins, only the *mcyE* gene was amplified in the samples, indicating the presence of cyanobacteria with the potential for hepatotoxin production in the raw water from July and in the treated water from June and July 2015 (Table 1). Unfortunately, the raw water filter from June 2015 was missing. The *mcyE*-positive fragments were sequenced and confirmed to belong to microcystin through comparison with the BLASTx database for proteins. Sequences were deposited in NCBI GenBank under the accession numbers MG914077-MG914079.

#### 2.1.3. Quantification of MC-LR by LC-ESI-MS/MS

**Figure 1:**
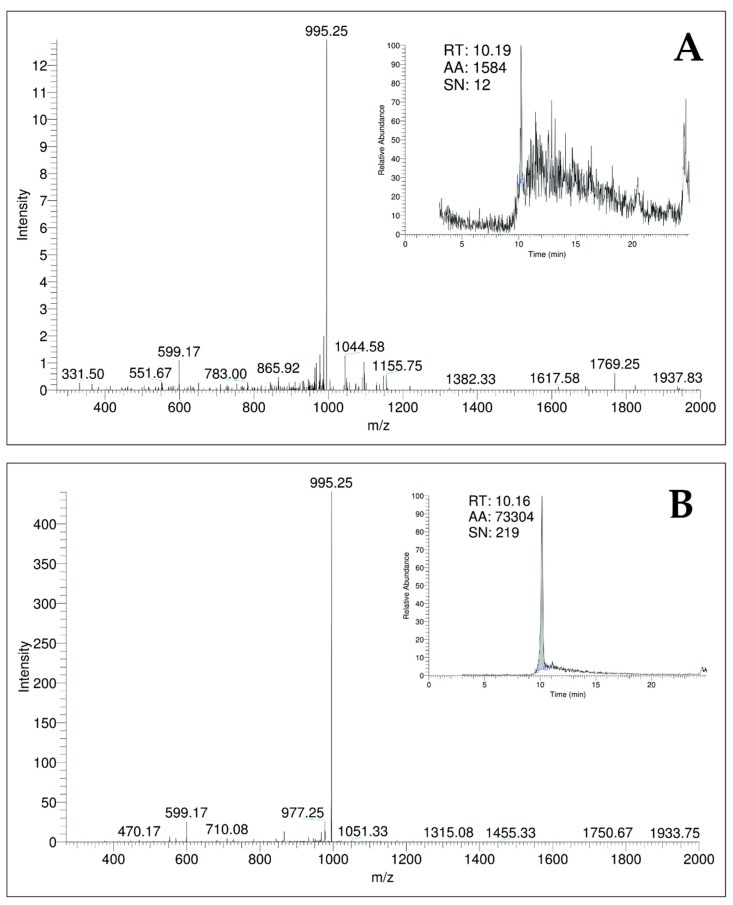
Mass spectra of MC-LR by LC-MS: (**A**) retention time and area of spiked sample of treated water from June 2015; (**B**) MC-LR standard.

The LC-MS analysis confirmed the presence of MC-LR in the treated water sample from June 2015 (Figure 1). The sample presented a retention time of 10.19 min and a signal at the m/z peak of 995.25, with m/z 599.17 and m/z 865.92 fragments. The toxin concentration was 0.026 µg L^−1^. The absence of MC-LR in the other samples, despite the positive ELISA and molecular biology results, may be explained by the preservation conditions and by the fact that the LC-MS analysis used an MC-LR standard and was therefore selective for MC-LR. On the other hand, ELISA is able to detect several microcystin congeners other than MC-LR (such as microcystin-RR and microcystin-YR) and does not indicate which congeners are present in a microcystin mixture [31].

### 2.2. Environmental Data

The physical, chemical, and biological characterizations of the two sampling sites (the raw water intake at the Amazon River and the treated water from the Macapá DWTP) during the one-year monitoring period are summarized in Table 2. Some parameters from the raw water, such as the river pH, which remained close to neutral (average = 6.6), the dissolved oxygen (DO) (average = 6.0 mg L^−1^), the water temperature (average = 28.8 °C), the electrical conductivity (EC) (average = 50.4 µS cm^−1^), total dissolved solids (TDS) (average = 24.3 mg L^−1^), and the water level (average = 3.1 m) did not differ significantly over the year. Other parameters, such as total coliforms (range = 2658.0–22,494.0 TC. 100 mL^−1^); *E. coli* (range = 68.0–6780.0 *E. coli*. 100 mL^−1^); flow (range = 89.3–279.9 m^3^ s^−1^); insolation (range = 1.3–10.6 h); and irradiation (range = 86.6–312.3 W m^−2^), varied substantially (Table 2).

Transparency, turbidity and chloride showed significant differences between May–December and January–April. Transparency (range = 12.0–36.5 cm) was higher from May to December (average = 31.4 cm) than from January to April (average = 13.1 cm); turbidity (range = 19.8-122.0 NTU) was 29.3 mg L^−1^ on average from May to December and increased to 107.6 mg L^−1^ on average from January to April. We observed the same pattern for chloride (range = 1.6–25.8 mg L^−1^); chloride was relatively low from May to December (average = 2.9 mg L^−1^) and increased in January–April (average = 20.4 mg L^−1^).

Regarding precipitation, the highest rainfall values occurred in February–April 2016 (average = 498.7 mm) and May 2015 (375.9 mm). The precipitation decreased in the trimester of June–August (average = 152.0 mm) until there was no rainfall in September–November and very low precipitation in December (26.6 mm). Lastly, the cyanobacteria average density was 2.14 × 10^2^ cells mL^−1^, with a density peak in July 2015 (1.09 × 10^3^ cells mL^−1^); notably, we also detected a major concentration of microcystin-LR (2.1 µg L^−1^) in the raw water intake in July 2015. The studied parameters of the treated water met the Brazilian legal guideline values for drinking water [20], except for iron and turbidity (Table 2).

### 2.3. Cyanobacteria Identification and Counting

Ten cyanobacteria taxa were identified in the microscopic monitoring of raw water from the Amazon River: *Limnothrix planctonica, Alkalinema pantanalense, Leptolyngbya* sp., *Pseudanabaena* sp., *Raphidiopsis* sp., *Anabaena* sp., *Dolichospermum* sp., *Geitlerinema splendidum*, *Cephalothrix lacustris*, and an unidentified cyanobacteria named Morphoespecies 1. The most frequent taxa were identified by polyphasic taxonomy [22], whereas the other taxa were identified only by morphology (microscopy). All of the cyanobacteria had filamentous forms.

**Figure 2:**
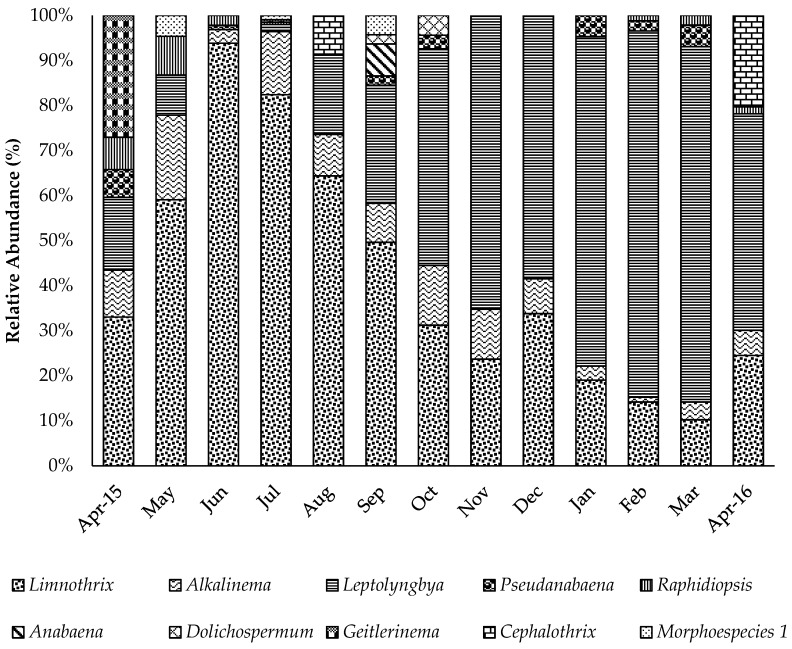
Relative abundance of cyanobacteria in the Amazon River raw water during the study period from April 2015 to April 2016.

The most abundant cyanobacteria in the Amazon River were *Limnothrix planctonica*, *Leptolyngbya* sp., and *Alkalinema pantanalense* (Figure 2). The first two taxa dominated the community composition, representing ≥ 50% of all specimens in the samples; *L. planctonica* was dominant during May–September, and *Leptolyngbya* sp. was dominant in November–March (Figure 2). *L. planctonica* and *A. pantanalense* occurred in 100% of the samples, *Leptolyngbya* sp. occurred in 92% of the samples, and the other species occurred in ≤ 50% of the samples. The highest values for cyanobacterial cell density were observed in July 2015 (1.09 × 10^3^ cells mL^−1^), whereas January presented the lowest cell density of 8.94 × 10 cells mL^−1^ (Table 2).

### 2.4. Linkages Between Environmental Factors, Cyanobacteria, and Cyanotoxins

To perform canonical correspondence analysis (CCA), a previous statistical analysis (forward selection) chose five variables that had more influence on the abundance of cyanobacteria and toxin concentration from a matrix of 26 environmental variables (Table 2): microcystin, TDS, chloride, dissolved oxygen, and transparency. These five variables were used to perform the CCA with the most abundant and frequent cyanobacteria: *L. planctonica*, *A. pantanalense*, *Leptolyngbya* sp., *Pseudanabaena* sp., *Raphidopsis mediterranea*, and *C. lacustris*).

**Figure 3:**
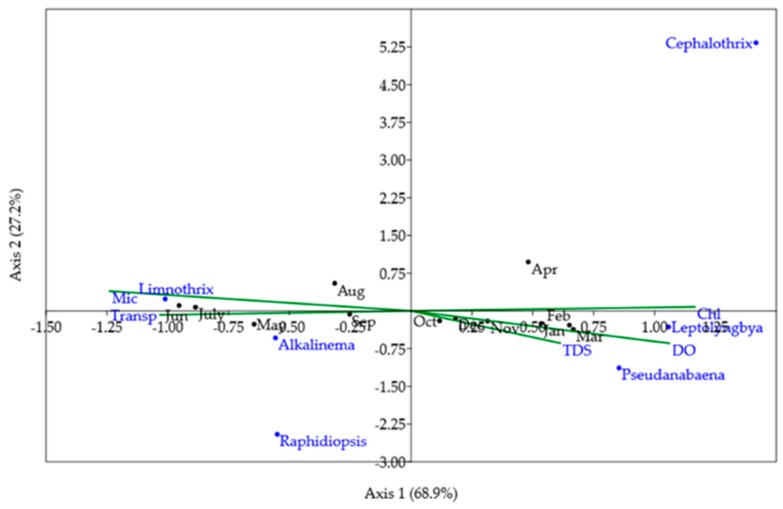
CCA triplot showing the sampling months, taxa, and environmental factors associated with MC-LR (Mic).

The ordination obtained in the CCA reflected the presence of MC-LR, the seasonal shifts between the dominant species found in the Amazon River (*Limnothrix planctonica* and *Leptolyngbya* sp.), and was also related to seasonal limnological changes, such as changes in transparency (Figure 3). The first two axes accounted for 96.15% of the variance (CA 1: 68.9%; CA2: 27.25%). The permutation test confirmed that the model was significant (*p* = 0.003), as were the first two CCA axes (*p* = 0.004; *p* = 0.016).

**Figure 4:**
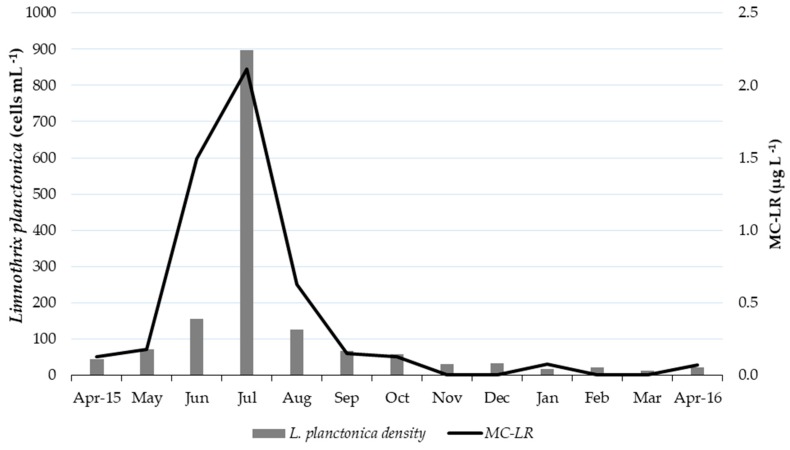
*Limnothrix planctonica* density and MC-LR concentration in the Amazon River from April 2015 to April 2016.

Microcystin (Mic) and chloride (Chl) showed the greatest variation in the first axis (CA1). Microcystin and transparency (Transp) were in the same quadrant as *Limnothrix*, *Alkalinema*, and the months of June, July, August, and September, showing a strong correlation between these variables. That is, the highest microcystin values were associated with the highest transparency values, observed in the period from May to September, mainly in June and July, the months presenting the highest *L. planctonica* densities (Figure 3 and Figure 4). *Alkalinema* abundance was related to the same parameters and months as *Limnothrix* abundance but presented lower average values.

On the right side of CA1 (Figure 3), chloride showed a strong correlation with the axis, followed by DO. This variation occurred between October and April and influenced the abundance of *Leptolyngbya* sp., which was high when the concentration of chloride and dissolved oxygen (DO) were highest. 

The significant correlation (*p* < 0.05) results of Spearman’s correlation analysis between all the studied factors are presented in Figure S2 (Supplementary Materials). The variables flow and *Limnothrix* showed a high correlation with MC-LR concentrations (Figure S2, Supplementary Materials).

## 3. Discussion

This study was the first to detect the presence of microcystin in the Amazon River. There have been few previous reports of this toxin in the Amazon region, specifically in the Tapajós River [32] and in the Bolonha Lake in Belém [33,34], but there has been no previous record of microcystin in the Amazon River. These results indicate the need to create a monitoring plan for the catchment area of the DWTP because cyanotoxin concentrations may increase over time and cause health risks to the local population. Although the observed cyanotoxin concentration seems relatively low, one must consider that the Amazon River has the largest volume of fresh water in the world, with a flow of approximately 219 m^3^.s^−1^ and two daily tides [29,35] at the sampling area, which is an estuarine zone. Therefore, the detection of MC-LR in the river mouth might also indicate that this cyanotoxin may be present in other sites of the river, degrading the water quality of this huge surface water source.

Some authors have previously commented that dilution is among the factors that can reduce microcystin toxicity in aquatic ecosystems [36,37]. However, pollutant dispersion does not occur homogeneously across the entire length or width of a river [23,24,38]. In the studied Amazon stretch, dispersion tends to be focused in riverbank plumes [23], especially under the effects of semi-diurnal tides that significantly influence pollutant plume transport and typically cover a larger area than the margins, including the raw water intake area of the DWTP, depending on the seasonal hydrodynamic conditions [24].

The ELISA and *mcyE* molecular marker results were positive for the samples from June and July 2015. However, the results of the two tests diverged in August, when only ELISA yielded a positive result (Table 1). This difference probably occurred as a result of cyanotoxin maintenance in the aquatic environment even after the disappearance of toxic cyanobacteria, a situation already reported in other studies [39]. The *mcyE* gene fragment found in the Amazon River samples is present in microcystin and nodularin-producing cyanobacteria (hepatotoxic strains) [40]. Microcystin is produced by various cyanobacterial species, whereas nodularin is currently produced by a few genera of cyanobacteria (*Nodularia*, *Nostoc*, and *Iningainema*) [41]. Using this marker, it is possible to quickly detect the presence of cyanobacteria with the potential for hepatotoxin production by extracting DNA and performing a PCR reaction [40].

Microcystins are extremely stable cyclic peptides that are resistant to chemical hydrolysis or oxidation at neutral pH, with high temperatures (even resisting boiling) [7], and can remain in a water body for an average of 90 to 120 days per meter of depth [42]. On the other hand, the degradation of microcystins may be enhanced by factors such as decreasing environmental pH [43]. Microcystins are inhibitors of protein phosphatases 1 and 2A. Acute exposure can lead to liver failure and death [6], and chronic exposure to small microcystin concentrations increases the risk of liver cancer. In regions of China (Jiangsu Province), liver cancer has increased in places with a microcystin concentration of 0.16 µg L^−1^ [44].

The microcystin concentration detected by LC/MS in the treated water sample from June (0.026 µg L^−1^) was below the guideline value for drinking water, that is, 1.0 µg L^−1^, provided by Brazilian Law [20] and recommended by the World Health Organization [19]. The MC-LR molecule was “atomized” on the LC/MS and showed a typical fragmentation pattern for an MC-LR, with a 995 m/z peak signal and 599 and 866 m/z fragments, as well as a retention time of approximately 10 min [5,45]. These MC-LR characteristics were compared to a standard to validate the analysis.

Although there are over 240 types of microcystins, few occur frequently and in high concentrations [11,36]. The MC-LR variant is among the most frequent and most toxic, with many toxicological data available, which is why it is the only variant with a WHO suggested reference value [19]. Exposure to microcystins usually occurs through water intake and recreation [19], which are predominant uses of the Amazon River waters in the studied stretch. It is common in Amazonian cities located on the banks of the Amazon River to use their waterfront for sports, bathing, fishing, and contemplation of nature. Usually, this area contains the city’s main squares, parks, and restaurants. These uses outside the aquatic environment may also be impaired by high concentrations of microcystins, as microcystins can be propagated by aerosols (airways), presenting health hazards because of toxin inhalation [46,47].

The following taxa were recorded in the months in which microcystin was observed: *Limnothrix planctonica*, *Leptolyngbya* sp., *Pseudanabaena* sp., *R. mediterranea*, *Alkalinema pantanalense*, *C. lacustris* and Morphospecies 1. *L. planctonica*, *Leptolyngbya* sp., and *A. pantanalense* had the highest densities (Figure 2). Of the cited genera, microcystin production has been recorded in *Limnothrix* sp. isolates (CENA 109 and CENA 110) obtained from a stabilization pond [48]. In addition, studies with the Australian *Limnothrix* AC0243 strain have shown that this genus is capable of producing a new toxin (Limnothrixin), for which no commercial kits are yet available [49,50,51]. This strain has characteristics of invasive species, as a strong competitor in dynamic environments such as estuaries [52].

MC-LR production has also been reported in *Leptolyngbya* cultures obtained in Brazil from a recreation reservoir in São Paulo [53]. Another case of MC-LR production was detected in *Leptolyngbya* strains obtained from a sewage treatment system (stabilization pond) [48]. There have also been records of the toxicity of this species abroad. *Leptolyngbya* sp. and *Geitlerinema* sp. were obtained from the biofilms of coral microorganisms (black band disease-BBD) in the Bahamas and Florida Keys, and HPLC/MS analysis and detection of the *mcyA* marker confirmed that the strains produced microcystin [54]. *Geitlerinema splendidum* CCIBt 3223 collected in Brazil also had the toxic effect of inhibiting acetylcholinesterase activity in rats; although this effect was irreversible, the strain did not produce MC-LR [55].

Regarding other taxa, *Pseudanabaena* has microcystin-producing strains [56,57] and is considered one of the most frequent toxin-producing genera [5]. *Raphidiopsis mediterranea* has presented strains producing other toxins [58]. The genus *Raphidiopsis* currently includes the genus *Cylindrospermopsis* and the species *Cylindrospermopsis raciborskii* [59], which is toxic, invasive, and widely distributed [5,60]. For the genera *Alkalinema* and *Cephalothrix*, we found no citations in the literature related to cyanotoxin production. However, *Alkalinema* [61] and *Cephalothrix* [62] are relatively new genera and still little studied. An interesting consideration is that *Alkalinema* exhibited similar behavior to *Limnothrix* throughout this study. Therefore, this taxon may also be responsible for the production of MC-LR.

Spearman’s correlation analysis showed a strong positive correlation between the concentration of microcystin and of the concentration of *Limnothrix* and the Amazon River flow, as well as a negative correlation between the concentration of microcystin and *Leptolyngbya* and turbidity. This indicates that MC-LR is produced even under high-flow conditions and consequently high velocity and turbulence [23,63]. These conditions make it difficult for photoautotrophic planktonic organisms to be maintained and grown in the water column [64,65,66].

In the case of the genus *Limnothrix*, which had a strong relationship with microcystin observed in both the correlation analysis and CCA, its success (peak growth) during the maximum flow of the Amazon River was facilitated by its morphological and physiological characteristics, such as the presence of aerotopes, which allow *Limnothrix* to migrate through the water column [67] and reduce its specific gravity [68], the high S/V ratio and cylindrical body shape of *Limnothrix*, which decrease sinking [69], and the planktonic and benthic habits of *Limnothrix*, which can adhere to substrates [49,52]. One of the factors that contributed to the dominance of this species was the low light penetration through the water body because of the presence of suspended materials. Rücker et al. [70] studied the factors controlling the dominance of *Limnothrix* species (*L. redekei*, *planctonica* and *amphigranulata*) in polymeric lakes (shallow lakes subjected to near-daily circulation) and concluded that light is a key factor in *Limnothrix* dominance because it grows efficiently in light-limited environments. Regarding temperature, the high water temperatures observed in this study seem to be ideal for *L. planctonica*. A study with a *Limnothrix* strain (AC0243) showed that the best growth results were obtained at temperatures of 35 °C [52]. Therefore, it is very probable that *L. planctonica* was the producer of MC-LR observed in this study.

Despite this strong evidence, it cannot be confirmed that *L. planctonica* or one of the cyanobacteria found in the monitoring of the DWTP was responsible for the production of MC-LR, since the cyanotoxin-related molecular marker (*mcyE*) was detected in an environmental sample that might have contained many other species, including picoplanktonic or nanoplanktonic cyanobacteria, that could not be observed with the inverted microscope. This problem has been reported in other studies, such as [53], and is due to both the usual 400x magnification of the inverted microscope analysis and the difficulty of processing whitewater river samples because of the large amount of solids that make planktonic organisms difficult to see [71,72].

To link a species to MC-LR production, it would be necessary to isolate the cyanobacteria and then search for the cyanotoxin-related marker in each cyanobacterial strain [30]. This process has already been performed with strains isolated from the water intake of the DWTP of Macapá, but none of these strains presented the potential for the production of microcystin, saxitoxin, anatoxin, or cylindrospermopsin [22]. In any case, the toxin (chemical) was detected by ELISA and LC-MS methods, indicating that some “still unknown” Amazonian cyanobacterial strain is producing such a metabolite.

Regarding the environmental variables, both raw and treated water were in accordance with the guideline values recommended by the Brazilian Law [20] and WHO guideline values. The microcystin detected in the raw water was lower in the treated water and within legal limits (<1.0 µg L^−1^). The cyanobacterial density was also always below 10,000 cells mL^−1^ in the raw water. Only iron (2.2 ± 1.26 mg L^−1^) and turbidity (55.5 ± 41 NTU) in the treated water exhibited values that did not comply with Brazilian Law [20]. The guideline value (GV) for iron in water at the DWTP exit is 0.3 mg L^−1^, but the legislation allows this level to be exceeded as long as the concentration is no higher than 2.4 mg L^−1^, among other criteria. The turbidity threshold is 5.0 uT (NTU) for the treated water distribution system, as there is no GV for the treated water at the DWTP exit. It is important to note that iron and turbidity were outside the organoleptic standards. Organoleptic parameters are related to the sensory stimuli that affect the acceptability of water for consumption (e.g., the poor acceptance of turbid water by the population) but are not directly linked to health risks [20].

CCA identified the environmental parameters that most influenced the occurrence and abundance of cyanobacteria and grouped the parameters with the greatest associations with each other. Since CCA is performed with a limited number of variables, Spearman’s correlation was also performed to understand the interactions of all the parameters with each other. The CA1 axis was divided by the two species with the highest densities (*L. planctonica* and *Leptolyngbya* sp.), both at an angle very close to the CA1 axis but at opposite ends.

The *Limnothrix* point on the CCA plot was very close to the transparency point, as well as the microcystin point. The density peak of this species occurred at a time when there was an increase in water transparency (Figure 3). Other changes of this period decreased in the monthly precipitation, turbidity, chloride concentration, EC, and ammonia (NH_3_) concentration. Interestingly, although *L. planctonica* is highly adapted to light-limited aquatic environments, the density of *L. planctonica* increased when conditions for light absorption improved. Photosynthesis can be much more efficient in such conditions of more transparent water [66], which was probably due to decreased rainfall that resulted in a less turbid environment with lower EC and chloride and ammonia concentrations. The decrease in chloride and ammonia occurred because these elements are carried from the city via runoff and enrich the river with nutrients [73].

A study on *Limnothrix* strain AC0243 growth at different light intensities (PAR, photosynthetically active radiation) of 0, 80, 160, 400 and 560 µE m^2^ s^−1^ showed an increase in cell concentrations under all light intensities, even at 0 µE m^2^ s^−1^, with added glucose. However, the highest concentration of cells occurred at an intermediate light intensity (160 µE m^2^ s^−1^) [52]. Most likely, the *L. planctonica* population of the Amazon River uses light most efficiently at the intensity that occurred from June to August, as a result of the interactions among factors such as the increased transparency, insolation and irradiation (Figure S2, Supplementary Materials).

At the other end of CA1, *Leptolyngbya* sp. was very close to the chloride, dissolved oxygen and *Pseudanabaena* sp. points. Chloride values increased with increasing turbidity, EC, and monthly precipitation. However, these parameters were negatively correlated with the water column transparency. This indicates that these species are well adapted to shading [74], which is also a result of the presence of clouds and rainfall causing increased runoff. Moreover, it can be inferred that *Leptolyngbya* sp. presents an even lower light tolerance than *Limnothrix* [52,75]. The authors of [75] studied the *Leptolyngbya* strain FLK1-C isolated from biofilms of microorganisms that cause coral BBD and found that the maximum photosynthetic activity was reached at low light levels (108 µE m^2^ s^−1^).

## 4. Conclusions

This study revealed the first detection of MC-LR in the Amazon River and provided information on the occurrence and density of cyanobacteria in this area. MC-LR was also detected in the treated water of the Municipality of Macapá.

Despite the detection of microcystin in the quarter of June to August 2015, at the same time as the *L. planctonica* density peak, the cyanotoxin concentration and cyanobacterial density were low throughout the study period. Both parameters were under the Brazilian and World Health Organization guideline values.

The fact that low densities of cyanobacteria cells can produce microcystin even in a water column mixing environment may help researchers to review the guideline values for cyanobacteria cell density in raw water.

The study analyses strongly indicate that *L. planctonica* strains produced the MC-LR detected in the waters of the Amazon River. Nevertheless, it is not possible to confirm this information because the chemical and molecular analyses were conducted on environmental samples (with the presence of several species).

The parameters that showed the highest association with the production of MC-LR and the increase in *L. planctonica* density were the increase in water transparency and flow. These results reinforce the indication that *L. planctonica* produced the detected toxin since this species is well adapted to the Amazon River conditions; for example, the density of *L. planctonica* can increase even in the maximum river flow. It is also important to note that *Limnothrix planctonica*, which already dominates the environment in the catchment area, tends to proliferate under rising temperatures because of climate change. Furthermore, increases in the density of these cyanobacteria may also be linked to the increment in nutrient concentrations because of the spread in organic loads resulting from uncontrolled urban sprawl without improvements in sanitation.

The characteristics of the registered cyanobacteria are very stable, so such toxin-producing events are likely to recur. However, the detection of cyanotoxin-producing events and the implementation of preventive actions will only be possible if adequate monitoring is available. Therefore, we conclude that it is necessary and urgent to initiate cyanobacterial and cyanotoxin monitoring to ensure the safety and health of the Macapá population in the medium and long term, given the low levels of basic sanitation in the municipality of Macapá. This can be extended to all the cities located in the Amazon River banks as most of them have a similar sanitary context as Macapá.

## 5. Materials and Methods

### 5.1. Study Site

**Figure 5:**
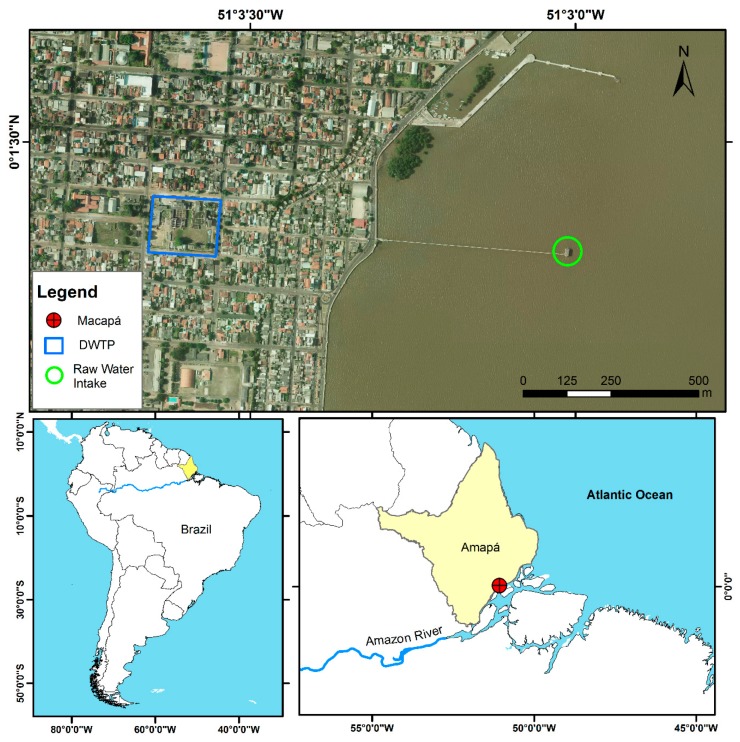
Study area in the Amazon River, Macapá, State of Amapá, Brazil. Reproduced with permission from Oliveira et al. [22], Phytotaxa, published by Magnolia Press, 2019.

This study was conducted at the Company of Water Supply and Sewage of Amapá (CAESA), specifically in the raw water intake situated in the Amazon River, North Channel (coordinates 0°1′19.50″ N 51°3′0.77″ W), and in the treated water at the drinking water treatment plant (DWTP) of Macapá, Brazil (0°1′22.72″ N 51°3′35.53″ W). The DWTP is 1000 m away from the raw water intake (Figure 5), and the water intake point is only 500 m away from the river margin [23,24]. This place is near the Amazon River mouth, an estuary area influenced by the seasonal variation in rainfall and semi-diurnal tides, which rise and fall twice daily [76].

### 5.2. Water Quality Sampling and Monitoring of Environmental Variables

Water quality sampling was carried out monthly from April 2015 to April 2016 at the raw water intake and at the exit from the treatment plant. Samples were collected during high tide to standardize the sampling conditions and to collect as little sediment as possible from the bottom of the river, a situation more favorable for cyanobacterial cell counting. For each sampling site (raw and treated water), we measured the physicochemical and microbiological parameters, as well as the cyanotoxins (Table 3). Notably, the focus of this paper was the monitoring of raw Amazon water. However, cyanotoxins were analyzed both pre- and post-treatment.

Physicochemical parameters (water temperature, pH, conductivity, dissolved oxygen, and TDS) were measured in situ using the equipment described in Table 3. Water transparency was also measured in the field with a Secchi disk. Furthermore, 500 mL of water samples were collected for each of the studied parameters: chemicals (NH_3_, NO_3_, PO4^3-^, SO_4_, Cl^−^), metals (Al^+3^, Fe), turbidity, coliforms, toxins for LC-MS, toxins for ELISA, and molecular biology (Table 3). Samples were preserved under refrigeration for laboratory analysis. For cyanobacterial counting, we collected 1 L of raw water preserved with 0.7% Lugol’s iodine in amber glass bottles stored in the dark [77].

The chemical and metal quantification was conducted using the spectrophotometric method with a DR 3900 spectrophotometer (Hach, Loveland, CO, USA), and turbidity was determined with a Turbidimeter AP2000 Policontrol following the recommendations of APHA [77]. Solar irradiation and insolation data for Macapá were provided by the National Institute of Space Research (INPE) [78], while precipitation and air temperature data were provided by the National Institute of Meteorology (INMET) [79]. Flow data were provided by an online database of the National Water Agency (ANA) [80]. Total and thermotolerant coliforms were estimated by the defined substrate technology (DST) with the Colilert^®^ commercial kit.

### 5.3. Cyanotoxins

To search for toxins in the water, two replicates of 500 mL samples of water before and after treatment were collected in triplicate (for ELISA, molecular screening, and LC-ESI-MS), filtered using GF/F fiberglass filters, and separated into dissolved and cellular fractions.

#### 5.3.1. Quantification of Cyanotoxins by ELISA

Filters were sonicated (5 times, 60 s, 60 Hz) on ice to extract the intracellular toxins. Then, samples were subjected to a freeze-thaw procedure three times and underwent a microscopy check and centrifugation. Dissolved and cellular fractions were analyzed in triplicate using the enzyme-linked immunosorbent assay (ELISA) Microcystin Plate Kit^®^ and Saxitoxin Plate Kit^®^ (Beacon, ME, USA). To confirm the results and avoid false positives, the positive samples were further analyzed by molecular screening of the GF/F filters and liquid chromatography-mass spectrometry (LC/MS).

#### 5.3.2. Molecular Screening for Cyanotoxins

The samples intended for molecular screening were preserved at −20 °C until the DNA extraction. Genomic DNA from all raw and treated water filters was extracted with the PureLink™ Genomic DNA kit (Invitrogen) using the Gram-negative bacteria lysis protocol according to the manufacturer’s recommendations. PCR amplification was carried out using the 16S rRNA gene for cyanobacteria detection, and molecular screening of genes involved in the production of cyanotoxins (microcystin, saxitoxin, anatoxin and cylindrospermopsin) was also conducted.

All PCR reactions were performed in a final volume of 20 µL, composed of 5x Green GoTaq^®^ Flexi Buffer, 25 mM MgCl_2_, 10 pmol of each forward and reverse primer (Table 4), 10 mM dNTP mix, bovine serum albumin (BSA) and 0.5 U of GoTaq^®^ DNA polymerase. The PCR reactions were performed in a Biometra T professional gradient Thermocycler (Biometra, Göttingen, Germany). The primers and positive controls used are described in Table 4. The PCR amplification conditions for each gene followed the protocol described in [39]. PCR products were visualized by agarose gel electrophoresis on 1.5% agarose gels stained with SYBR^®^ Safe.

The positive results for the fragments of genes involved in the production of microcystin were purified with the NucleoSpin^®^ Gel and PCR Clean-up kit (Macherey-Nagel, Düren, Germany) and sequenced bidirectionally through Sanger sequencing by the company GATC Biotech (Constance, Germany). The sequences were then aligned with Geneious (version 8.1, Auckland, New Zealand) and compared with sequences deposited in the GenBank database using BLASTx (Basic Local Alignment Search Tool) to confirm the toxin identification. Sequences were deposited in NCBI GenBank under the accession numbers MG914077-MG914079.

#### 5.3.3. Quantification of MC-LR by LC-ESI-MS

The positive samples by ELISA and molecular screening were also examined using liquid chromatography coupled with mass spectrometry (LC-MS) to confirm the presence of MC-LR.

The dissolved samples were processed through solid-phase extraction (SPE) to clean the sample and concentrate the cyanotoxins. The cellular fractions were sonicated with MeOH 50% (5 times, 60 s, 60 Hz) on ice to extract the intracellular toxins, followed by freezing and thawing three times, a microscopy check, and centrifugation. Then, the supernatant of the cellular fractions and the dissolved samples were lyophilized. The obtained solid was diluted 9× to avoid matrix effects.

The LC-MS system used to quantify the MC-LR was a Liquid Phase Chromatograph Finnigan Surveyor (Thermo Scientific, San Jose, CA, USA) coupled with a spectrometry detector (MS Mass LCQ FleetTM ion trap) with an electrospray (ESI) interface, including a Surveyor LC pump, a Surveyor autosampler, and a Surveyor photoelectric diode-array (PDA) detector. The program used for data acquisition and processing was XcaliburTM version 2 (Thermo Scientific, San Jose, CA, USA). The mass spectrometer was operated in full scan mode. The capillary voltage and tube lens were maintained at 22 and 120 kV, respectively; the spray voltage was 5.5 kV. Nitrogen was used as a sheath and auxiliary gas. The sheath gas flow rate was set at 80 (arbitrary units), and the auxiliary gas was set at 10. The capillary temperature was held at 350 °C. Helium was used as a collision gas in the ion trap at a pressure of 3 bar.

Separation was achieved on a C18 Hypersil Gold column (100 × 4.6 mm I.D., 5 μm, ThermoScientific, Waltham, MA, USA) kept at 25 °C, with a flow rate of 0.7 mL/min. The injected volume was 10 μL in loop partial mode. Samples were injected in positive polarity mode in full scan (270–2000 m/z). The standards and samples were injected in duplicate, and a blank and two standards of different concentrations were introduced to each set of six samples. The standard solution of MC-LR was purchased from DHI LAB Products (Hørsholm, Denmark, Batch nº MC-LR-110), with a concentration of 11.026 μg/mL. The system was calibrated using seven dilutions of the standard solution of MC-LR (between 8.5 and 180 μg/L) diluted in 50% acetonitrile (ACN).

A gradient elution was used with mobile phase A, ACN and B water, both acidified with 0.1% formic acid (55% A and 45% B at 0 min, 90% A and 10% B at 12 min, 100% A at 12.5 min, 100% A at 15 min, 45% A and 55% B at 15.01 and 25 min). Under these conditions, the MC-LR retention time was 10.16 min, and the LOD and LOQ were 5.7 μg/L and 8.5 μg/L, respectively.

Samples were analyzed using the mass-to-charge ratio (m/z) transition of 995 > 599 at 35 eV collision energy. The MC-LR transition was monitored for 1 microscan time. The precursor ion (m/z 995) and MC-LR reference fragment ions with m/z values of 375, 553, 599, 866, and 977 were monitored in the MS/MS mode to validate the presence of the toxin.

### 5.4. Cyanobacteria Identification and Counting

Cyanobacterial cell density was determined using the Utermöhl sedimentation method in 5 mL Utermöhl chambers. These chambers were chosen because 5 mL was the maximum volume that allowed cell visualization because of the high amount of solids in the Amazon River. Because we could not use larger volumes for cell visualization, we counted the cells across the whole chamber base. To obtain the final density (cells mL^−1^), cells were multiplied by 0.2 (1/5 = one whole chamber divided by the decanted volume 5 mL) [77]. A magnification of 400× was used to enumerate the cells under a Zeiss Axiovert A1 inverted microscope with a Zen Lite 2012 image capture system.

The cyanobacterial morphological identification was realized with specialized literature [60,67,87] and recent taxonomic papers describing new genera [61,62]. The most frequent and abundant species were isolated and underwent taxonomic confirmation by molecular methods (16S rRNA gene sequences) and phylogeny [22].

### 5.5. Statistics

A CCA was used to infer the influence of environmental factors on the abundance of cyanobacteria and toxin concentrations. To perform the CCA, we used data on the relative abundance of cyanobacteria [88]. Species with a density ≤ 10% of the total density were excluded from the analysis. First, we performed ln (x + 1) transformation of biological and environmental data, followed by the selection of environmental variables using forward selection in the “ordistep” function of the vegan package, R 3.4.3 software [89].

Subsequently, we calculated the inflation factor (VIF) to exclude multicolinear variables, eliminating the variables with VIF ≥ 15 [90]. This selection aimed at removing irrelevant explanatory variables in the analysis, highly correlated factors, and variables with relatively little variation [88]. After the screening, CCA was performed in Past 3.0 software with five environmental variables (microcystin, TDS, chloride, dissolved oxygen, and transparency) and the most common cyanobacteria species (6) detected during the 12 months of monitoring beginning in May 2015. Some parameters were not sampled in April 2015, so this month was not considered.

In addition to the CCA, we performed Spearman’s correlation analysis between all the studied factors to improve the interpretation of the results.

## Figures and Tables

**Table 1:** Results of the detection of microcystin by molecular screening (*mcyE*) and ELISA (MC-LR).

Sampling	Raw Water		Treated Water	
	*mcyE*	ELISA	*mcyE*	ELISA
Apr15	-	-	-	-
May	-	-	-	-
Jun	* ^1^	+	+	+
Jul	+	+	+	+
Aug	-	+	-	-
Sep	-	-	-	-
Oct	-	-	-	-
Nov	-	-	-	-
Dec	-	-	-	-
Jan	-	-	-	-
Feb	-	-	-	-
Mar	-	-	-	-
Apr16	-	-	-	-

^1^ Missing filter; + positive result; - negative result.

**Table 2:** Mean and minimum–maximum values of the chemical, physical, and biological parameters of the Amazon River raw water and treated water from the drinking water treatment plant (DWTP) of Macapá.

Parameter	Raw Water		Treated Water		Brazilian Guideline Values for Drinking Water [20]
	Average ± SD	Min–Max	Average ± SD	Min–Max	
pH	6.6 ± 0.28	6.0–7.2	6.0 ± 0.30	5.4–6.7	In accordance
Dissolved Oxygen (mg L^−1^)	6.0 ± 0.71	4.8–6.8	4.2 ± 0.20	3.8–4.5	- ^1^
Microcystin– LR (µg L^−1^)	0.4 ± 0.69	0.0–2.1	0.01 ± 0.03	0.0–0.1	In accordance
Nitrate (mg L^−1^)	0.4 ± 0.83	0.0–2.5	0.9 ± 1.30	0.02–4.5	In accordance
Ammonia (mg L^−1^)	0.7 ± 0.54	0.05–1.6	0.1 ± 0.05	0.0–0.2	In accordance
Orthophosphate (mg L^−1^)	0.3 ± 0.44	0.02–1.6	0.13 ± 0.09	0.05–0.4	- ^1^
Chloride (mg L^−1^)	8.7 ± 9.17	1.6–25.8	3.8 ± 1.70	1.8–6.8	In accordance
Sulfate (mg L^−1^)	1.8 ± 1.48	0.0–4.0	14.0 ± 3.60	9.0–21.0	In accordance
Aluminum (mg L^−1^)	0.1 ± 0.07	0.03–0.3	0.11 ± 0.06	0.006–0.2	In accordance
Iron (mg L^−1^)	2.2 ± 1.26	0.89–5.4	0.7 ± 1.10	0.07–3.9	Above the guideline value 0.3
Transparency (cm)	25.3 ± 9.60	12.0–36.5	-	-	- ^1^
Euphotic Zone (cm)	75.9 ± 28.90	36.0–109.5	-	-	- ^1^
Turbidity (NTU)	55.5 ± 41.0	19.8–122.0	9.8 ± 13.50	1.4–48.9	Above the guideline value 5.0
Total Dissolved Solids (ppm)	24.3 ± 4.41	20.0–30.0	31.2 ± 8.20	20.0–40.0	In accordance
Electrical Conductivity (µS cm^−1^)	50.4 ± 7.81	40.0–60.0	68.2 ± 10.70	50.0–80.0	- ^1^
Water Temperature (°C)	28.8 ± 1.70	25.0–30.3	28.9 ± 1.70	24.4–30.7	- ^1^
Minimum Air Temperature (°C)	24.3 ± 0.80	22.7–25.7	24.3 ± 0.80	22.7–25.7	- ^1^
Maximum Air Temperature (°C)	33.2 ± 2.00	28.9–35.3	33.2 ± 2.00	28.9–35.3	- ^1^
Insolation (h)	7.7 ± 3.40	1.3–10.6	7.7 ± 3.40	1.3–10.6	- ^1^
Irradiation (W m^−2^)	241.9 ± 75.60	86.6–312.3	241.9 ± 75.60	86.6–312.3	- ^1^
Daily Rain Precipitation (mm)	5.3 ± 7.60	0.0–22.4	5.3 ± 7.60	0.0–22.4	- ^1^
Monthly Rain Precipitation (mm)	206.0 ± 209.00	0.0–528.2	206.0 ± 209.00	0.0–528.2	- ^1^
Water Level (m)	3.1 ± 0.20	2.9–3.4	3.1 ± 0.20	2.9–3.4	- ^1^
Flow (m^3^ s^−1^)	177.376 ± 72.82	89.3–279.9	177.376 ± 72.82	89.3–279.9	- ^1^
Total Coliforms (TC/100 mL)	12838.7 ± 7239.23	2658.0–22494.0	0.0	0.0	In accordance
*E. coli* (*E. coli*/100 mL)	1082.7 ± 1832.57	68.0–6780.0	0.0	0.0	In accordance
Cyanobacteria (cells mL^−1^)	214.7 ± 277.81	89.4–1090.0	-	-	In accordance

^1^ No guideline values for drinking water purposes [20].

**Table 3:** Units of measurement of the parameters used, their methods, and analysis equipment.

Type	Parameter	Unity	Method/Equipment
Chemical	Ph	pH	pH-meter OrionStar A121 Thermoscientific
	Dissolved Oxygen	mg L^−1^	YSI 550 A DO probe
	Microcystin- LR	µg L^−1^	ELISA, molecular biology, LC-ESI-MS/MS
	Nitrate	mg L^−1^	Reduction Cadmium/Spectrophotometer
	Ammonia	mg L^−1^	Nessler/Spectrophotometer
	Orthophosphate	mg L^−1^	Phosver3/Spectrophotometer
	Chloride	mg L^−1^	Mercuric Thiocyanate/Spectrophotometer
	Sulfate	mg L^−1^	Method Sulfaver/Spectrophotometer
	Aluminum	mg L^−1^	Aluver/Spectrophotometer
	Iron	mg L^−1^	Ferrover/Spectrophotometer
Physical	Transparency	Cm	Secchi Disk
	Euphotic Zone	Cm	Secchi Disk x 3.0
	Turbidity	NTU	Turbidimeter AP2000 Policontrol
	Total Dissolved Solids	Ppm	Portable EC, TDS and Temperature meter HI8730 Hanna
	Electrical Conductivity	µS cm^−1^	Portable EC, TDS and Temperature meter HI8730 Hanna
	Water Temperature	°C	pH-meter OrionStar A121 Thermoscientific
	Air Temperature Min	°C	INPE
	Air Temperature Max	°C	INPE
	Insolation	H	INPE
	Irradiation	W m^−2^	INPE
	Daily Rain	Mm	Weather station/INMET
	Monthly Rain	Mm	Weather station/INMET
	Water Level	M	Tidal tables
	Flow	m^3^ s^−1^	HidroWeb/ANA
Microbiol.	Total Coliforms	TC 100 mL^−1^	Chromogenic Substrate
	*E. coli*	*E. coli* 100 mL^−1^	Chromogenic Substrate
	Cyanobacteria	Cells mL^−1^	Utermöhl

**Table 4:** Primers used to detect cyanobacteria and gene clusters of the cyanotoxins microcystin, saxitoxin, anatoxin, and cylindrospermopsin.

Gene	Primer	Primer Sequence 5’-3’	Size (bp)	Reference	Positive Control
**16S**	CYA106F/CYA781R	CGGACGGGTGAGTAACGCGTGA GACTACTGGGGTATCTAATCCCATT	675	[81]	-
**16S**	CYA359F/1494R	GGGGAATYTTCCGCAATGGG TACGGCTACCTTGTTACGAC	1135	[81,82]	-
**mcyA**	CD1F/CD1R	AAAATTAAAAGCCGTATCAAA AAAAGTGTTTTATTAGCGGCTCAT	297	[83]	LEGE91339-*Microcystis aeruginosa*
**mcyE**	HEPF/HEPR	TTTGGGGTTAACTTTTTTGGGCATAGTC AATTCTTGAGGCTGTAAATCGGGTTT	472	[40]	LEGE91339-*Microcystis aeruginosa*
**sxtI**	SXTI 682F/SXTI 877R	GGATCTCAAAGAAGATGGCA GCCAAACGCAGTACCACTT	100	[84]	LMECYA 040-*Aphanizomenon gracile*
**anaC**	anaCF/anaCR	TCTGGTATTCAGTCCCCTCTAT CCCAATAGCCTGTCATCAA	366	[85]	LEGE X-002-*Anabaena* sp.
**pks**	M4/M5	GAAGCTCTGGAATCCGGTAA AATCCTTACGGGATCCGGTGC	535/ 540	[86]	LEGE 97,047-*Cylindrospermopsis raciborskii*
**ps**	M13/ M14	GGCAAATTGTGATAGCCACGAGC GATGGAACATCGCTCACTGGTG	511/ 534	[86]	LEGE 97,047-*Cylindrospermopsis raciborskii*

## Supplementary Materials

The following materials are available online at https://www.mdpi.com/2072-6651/11/11/669/s1. Figure S1: Intracellular and extracellular fractions of microcystin from raw and treated water of the DWTP of Macapá. Figure S2: Spearman correlation matrix with all environmental variables from the study (described in Table 1). Red = negative correlation; blue = positive correlation; squares indicate *p* < 0.05.
