# Supplementary material for: First Detection of Microcystin-LR in the Amazon River at the Drinking Water Treatment Plant of the Municipality of Macapá, Brazil
Source: Toxins (Basel). 2019 Nov 15;11(11):669. doi: 10.3390/toxins11110669 (PMC6891726; doi:10.3390/toxins11110669)
Supplement: Supplementary file 1 [file toxins-11-00669-s001.pdf]

Supplementary Materials: First Detection of Microcystin-LR in the Amazon River at the Drinking Water Treatment Plant of the Municipality of Macapá, Brazil

Elane D. C. Oliveira, Raquel Castelo-Branco, Luís Silva, Natalina Silva, Joana Azevedo, Vitor Vasconcelos, Silvia Faustino and Alan Cunha

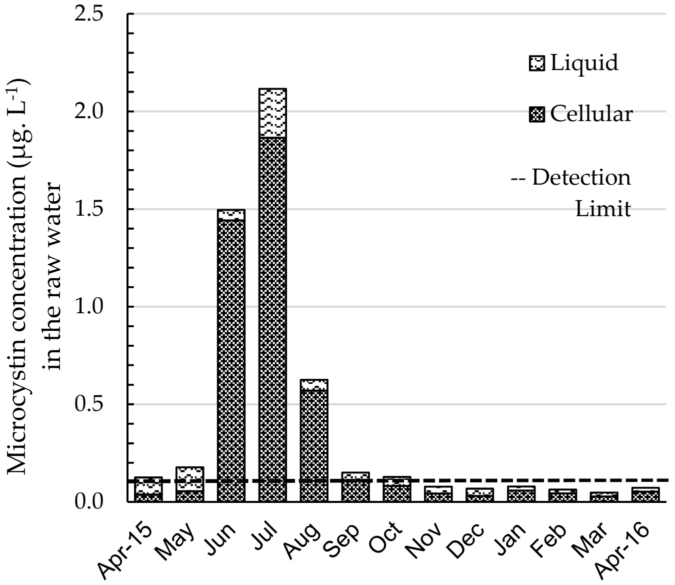

Figure S1. Intracellular and extracellular fractions of microcystin from raw and treated water of the DWTP of Macapá.

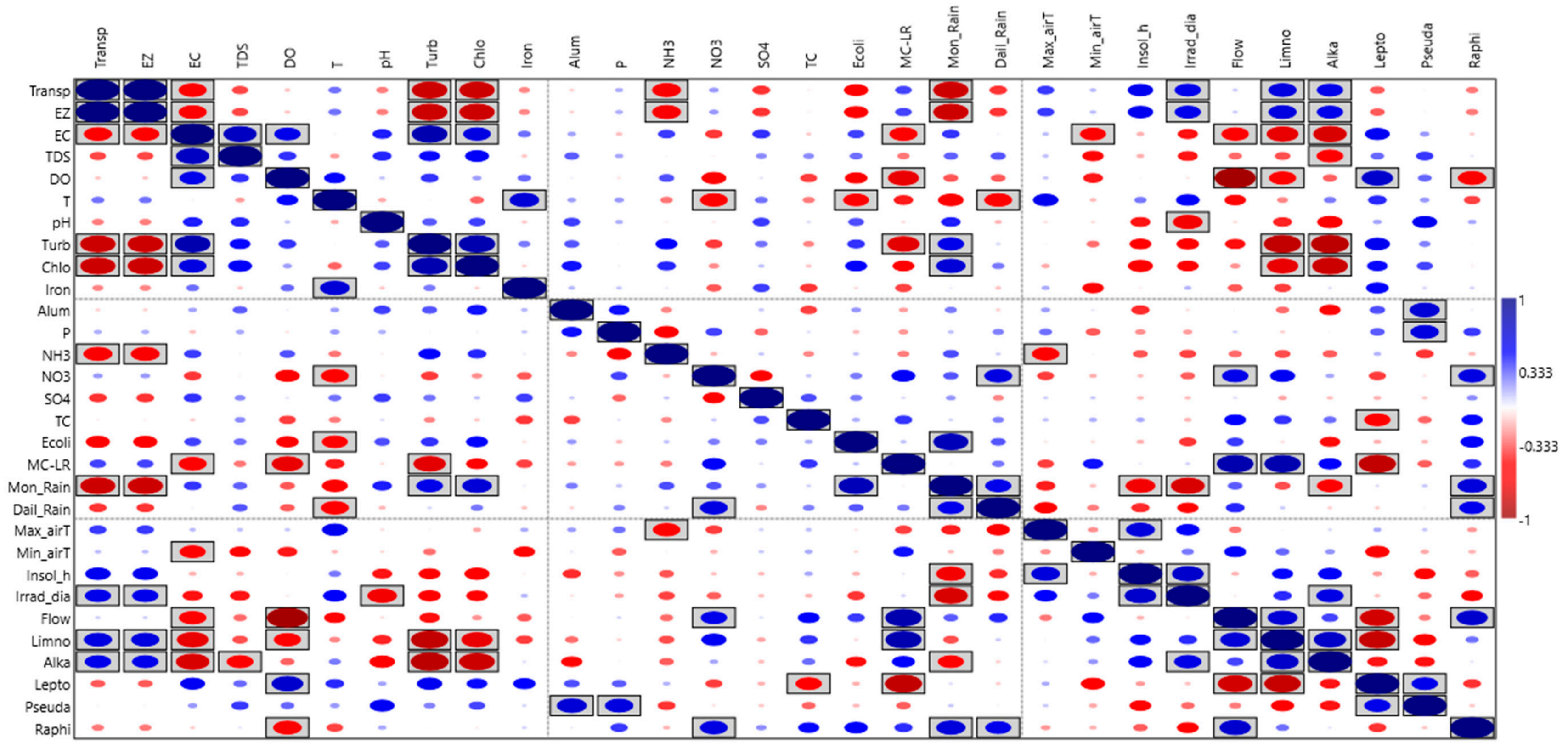

Figure S2. Spearman correlation matrix with all environmental variables from the study (described in Table 1). Red = negative correlation; blue = positive correlation; squares indicate $p < 0.05$. Legend: Transp- Transparency; EZ- Euphotic Zone; EC- Electrical Conductivity; TDS- Total Dissolved Solids; DO- Dissolved Oxygen; T- Water Temperature; pH; Turb- Turbidity; Chlo- Chloride; Iron; Alum- Aluminum; P- Orthophosphate; NH_3- Ammonia; NO_3- Nitrate; SO_4- Sulfate; TC-Total Coliforms; Ecoli- *E.coli*; MC-LR- Microcystin- LR; Mon_Rain-Monthly Rain Precipitation; Dail_Rain-Daily Rain Precipitation; Max_airT- Maximum Air Temperature; Min_airT- Minimum Air Temperature; Insol- Insolation; Irrad- Irradiation; Flow; Limno-*Limnothrix*; Alka- *Alkalinema*; Lepto- *Leptolyngbya*; Pseuda- *Pseudanabaena*; Raphi- *Raphidiopsis*.
